# Transcriptomic Profiling Unravels Novel Deregulated Gene Signatures Associated with Acute Myocardial Infarction: A Bioinformatics Approach

**DOI:** 10.3390/genes13122321

**Published:** 2022-12-09

**Authors:** Sanjay Kumar, Chun-Ming Shih, Lung-Wen Tsai, Rajni Dubey, Deepika Gupta, Tanmoy Chakraborty, Naveen Sharma, Abhishek Vikram Singh, Vishnu Swarup, Himanshu Narayan Singh

**Affiliations:** 1 Department of Life Science, Sharda School of Basic Sciences and Research, Sharda University, Knowledge Park-III, Greater Noida 201310, India; 2 Division of Cardiology, Department of Internal Medicine, Taipei Medical University Hospital, Taipei 111031, Taiwan; 3 Department of Internal Medicine, School of Medicine, College of Medicine, Taipei Medical University, Taipei 111031, Taiwan; 4 Department of Medicine Research, Taipei Medical University Hospital, Taipei 111031, Taiwan; 5 Department of Information Technology Office, Taipei Medical University Hospital, Taipei 11031, Taiwan; 6 Graduate Institute of Data Science, College of Management, Taipei Medical University, Taipei 11031, Taiwan; 7 Department of Neurology, All India Institute of Medical Sciences, New Delhi 110029, India; 8 Department of Chemistry and Biochemistry, Sharda School of Basic Sciences and Research, Sharda University, Knowledge Park-III, Greater Noida 201310, India; 9 Biomedical Informatics Division, Indian Council of Medical Research, New Delhi 110029, India; 10 Department of Cardiology, Infinity Care Hospital, Varanasi 221002, India; 11 Department of System Biology, University of Columbia Irving Medical Center, New York, NY 10032, USA

**Keywords:** acute myocardial infarction, differential expressed genes, weighted gene correlation network analysis, NOTCH1 signaling, cardiovascular disease

## Abstract

Acute myocardial infarction (AMI) is a severe disease with elevated morbidity and mortality rate worldwide. This is attributed to great losses of cardiomyocytes, which can trigger the alteration of gene expression patterns. Although several attempts have been made to assess the AMI biomarkers, to date their role in rescuing myocardial injury remains unclear. Therefore, the current study investigated three independent microarray-based gene expression datasets from AMI patients (n = 85) and their age–sex-matched healthy controls (n = 70), to identify novel gene signatures that might be involved in cardioprotection. The differentially expressed genes (DEGs) were analyzed using ‘GEO2R’, and weighted gene correlation network analysis (WGCNA) was performed to identify biomarkers/modules. We found 91 DEGs, of which the number of upregulated and downregulated genes were 22 and 5, respectively. Specifically, we found that the deregulated genes such as ADOR-A3, BMP6, VPS8, and GPx3, may be associated with AMI. WGCNA revealed four highly preserved modules among all datasets. The ‘Enrichr’ unveiled the presence of miR-660 and STAT1, which is known to affect AMI severity. Conclusively, these genes and miRNA might play a crucial role the rescue of cardiomyocytes from severe damage, which could be helpful in developing appropriate therapeutic strategies for the management of AMI.

## 1. Introduction

Despite the advancement in prevention and therapy, cardiovascular diseases (CVD) remain a major cause of human deaths globally. Approximately 17.9 million people die each year due to CVDs, which is estimated to be 31% of total deaths worldwide [1]. It has been further reported that 4 out of 5 CVD-associated deaths occur due to heart attacks in the population under the age of 70 years, and myocardial infarction (MI) is one of the major etiology. The elevating global incidence of acute myocardial infarction (AMI) includes multifactorial, such as environment, hereditary, and lifestyle. The pathology of AMI includes atherosclerotic rupture causing a monocyte and macrophage inflammatory cascade, thrombus development, and platelet aggregation. Consequently, transport of oxygen via the coronary artery is suppressed, leading to inhibited myocardial oxygenation [2]. Further, the mitochondrial inability to synthesize ATP activates the ischemic cascade, resulting in endocardial apoptosis (cell death) or MI. The AMI patients not only suffer from severe complications such as cardiac rupture, arrhythmia, and ventricular aneurysms, but also bear a mental and financial burden on the family [3]. Early diagnosis, by identifying the risk factors, preventing adverse cardiac events, and ensuring their timely treatment, is always a primary focus for clinicians in the management of AMI. There are several risk factors, such as age, sex, lifestyle, hypertension, dyslipidemia, diabetes, hypercholesterolemia, smoking, obesity, and genetic parameters, which might contribute to the majority of myocardial infractions, but further research is required to reveal additional risk factors [4,5]. Although coronary angiography has been considered as the “gold standard” for the detection of AMI, this method is invasive, expensive, and could be risky to patients [5,6]. Despite such advancement, the exact molecular mechanisms involved in pathophysiological processes for AMI development and progression have not been completely elucidated.

Currently, the reduced oxygen supply in the heart, pharmaceutical drugs, intravenous thrombolysis, and percutaneous coronary interventions are being utilized as important approaches to combat AMI [7]. However, these therapeutic strategies cause many complications during the treatment process, such as enhanced episode of bleeding due to antithrombotic drugs [8]. Comparatively, percutaneous coronary intervention (PCI) is more efficient therapy for acute ST-segment elevation myocardial infarction [9]. Overall, earlier detection of AMI is very important for patients to receive timely treatment.

A microarray study in AMI demonstrated that many genes, associated with different functions such as lipid/glucose metabolism, platelet function and atherosclerotic plaque stability, were altered. Upregulated genes such as SOCS3 and FAM20 were also observed in the first few days of AMI [10]. Furthermore, RNA profiling in AMI patients has shown that genes involved in chemotaxis, IL (interleukin)-6, and NF-κB (nuclear factor-κB) signaling were upregulated [11].

There are several reports on the bioinformatics analyses of single microarray data, demonstrating important genes related to the diagnosis or prognosis of AMI [12]. However, an integrated analysis of two or more microarray assay findings (i.e., microarray datasets), allows the analysis of differentially expressed genes (DEGs) among a large number of samples with similar conditions that leads to more robust and reliable results. Here, we used 155 samples based on microarray gene expression data to identify DEGs and their involvement in a signaling pathway associated with the pathogenesis of AMI and cardioprotection.

## 2. Materials and Methods

### 2.1. Microarray Datasets

Genome-wide gene expression datasets (n = 3), generated by a microarray study, were downloaded from the National Centre of Biotechnology Information (NCBI) Gene Expression Omnibus (GEO) database (https://www.ncbi.nlm.nih.gov/). Datasets having AMI patients (who had not received any treatments) and control samples were selected for this study. These three datasets included GSE66360, GSE29532 and GSE62646, which had 85 AMI patients and 70 age–sex-matched healthy controls. The healthy controls were also matched with confounders such as smoking, hypertension, and LDL (low-density lipoproteins). The number of patients/controls, sample type, and the platform of microarray experiments for each of the datasets utilized are represented in Table 1.

### 2.2. Screening of Differentially Expressed Genes (DEGs)

The GEO2R web tool was used to extract gene expression and control samples from patients. It uses GEOquery and limma (Linear Models for Microarray Analysis) R packages from the Bioconductor project. The GEOquery R package parses GEO data into R data structures by other Bioconductor/R packages. The GEO2R extracts pre-calculated expression values of the genes from the series matrix files deposited in the raw data of the corresponding studies. To calculate gene expression values where a single gene has multiple expression values, the average gene expression value was taken into consideration. The gene symbols of the probes of each dataset were mapped to the corresponding GEO Platform Accession Number information.

To calculate the differential expression value between patients and controls, the mean differences in gene expression were considered. Samples from all datasets were combined to get an overall expression. The significance of the differential expression was computed using an unpaired Student’s t-test. The genes with fold change > 1.6 with *p* value < 0.05 were considered as significantly altered genes.

### 2.3. Weighted Gene Co-Expression Network Analysis (WGCNA) and Module Identification

The weighted gene co-expression network among the DEGs was constructed using WGCNA R package [13]. The pickSoftThreshold function of the package was used for selecting parameters to identify scale free topology in the simulated DEG data. The unsupervised hierarchical clustering was used to detect the modules that is composed of densely interconnected genes. The modules are represented by the branches of the hierarchical clustering dendrogram, constructed using the branch cutting method. The gene information from each module was extracted and visualized using the network of eigengenes.

### 2.4. Pathway Enrichment Analysis of Turquoise Module

A KEGG (Kyoto Encyclopaedia of Genes and Genomes) based pathway enrichment analysis of turquoise genes module was performed using the STRING database. The false discovery rate (FDR) < 0.05 was considered to indicate a statistically significant difference between pathways. Furthermore, the ReactomeFIViz (version 2018), a cytoscape plugin (version 3.7.1), was used to find the network patterns of transcription factors extracted from the STRING database. It was used to visualize hit pathways using manually laid-out diagrams directly in Cytoscape, and investigate the functional relationships among genes.

### 2.5. Enrichment of TFs and microRNAs in Turquoise Module

The Enrichr webtool [14] was exploited to extract miRNA targeting the module genes. Significantly (*p* < 0.05) enriched miRNAs were extracted. Additionally, the information on protein–protein interactions (PPI) of transcription factors (TFs) was fetched from the Enrichr webtool.

## 3. Results

**Figure 1:**
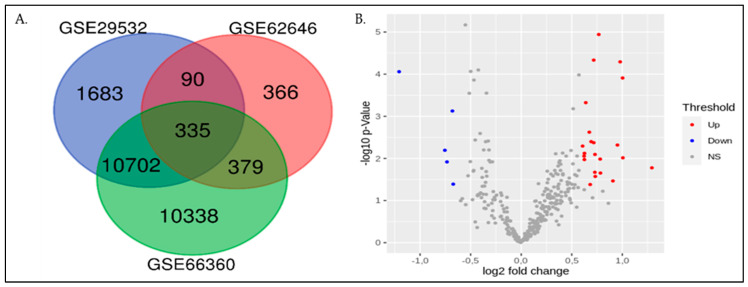
The expression of genes among three different data sets. (**A**) Venn diagram of gene expression in the three datasets: GSE29532 (blue), GSE62646 (red) and GSE66360 (green). (**B**) Analysis of DEGs in datasets GSE62646, GSE29532, and GSE66360. The negative log10-transformed *p*-values of the significant level were plotted against the log ratios (log2FC). The red, blue, and grey colors indicate the upregulated, downregulated, and insignificant levels of gene expression. DEGs: Differentially expressed genes; NS: Not significant.

Differential expression analyses of 85 patients and 70 controls were performed on the microarray datasets (Table 1). The number of genes 12,810, 1170, and 21,754 were extracted from the expression datasets GSE29532, GSE62646, and GSE66360, respectively. We found 11,037 common genes between GSE29532 and GSE66360 datasets, whereas 714 genes between GSE62646 and GSE66360. In addition, 425 genes were common between GSE62646 and GSE29532 datasets. Finally, 335 annotated genes were common among all three datasets (Figure 1A). Out of 335 annotated genes, 27 genes were significantly altered (Fold change > 1.6; *p*-value < 0.05), revealing 22 upregulated and 05 downregulated genes (Figure 1B; Supplementary Table S1).

**Figure 2:**
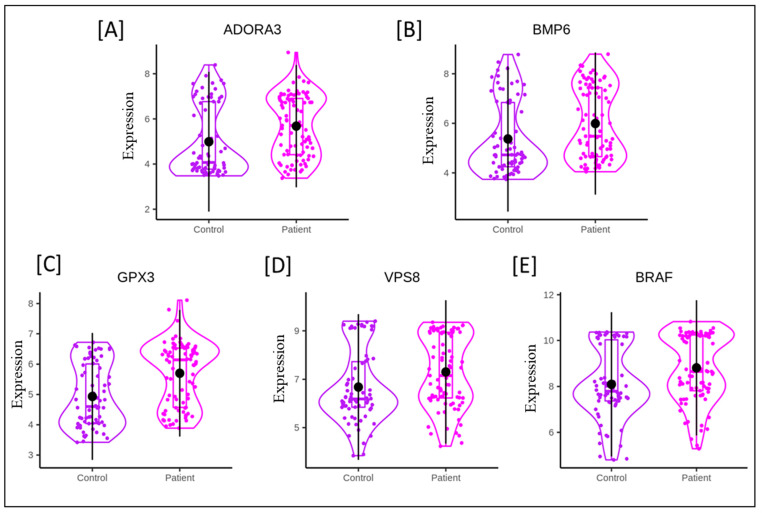
Violin plots of five DEGs (*p* < 0.05) obtained in pre-processing of pooled microarray datasets. (**A**) ADORA3, (**B**) BMP6 (**C**) GPX3 (**D**) VPS8, and (**E**) BRAF genes. The expression value (on the *y*-axis) was calculated in the terms of Log2 scale.

Some of the DEGs identified in the present analysis have been plotted in Figure 2 and presented in Table 2. The deregulated expression levels of these genes, such as ADOAR3, BMP6, GPX3, VPS8, BRAF, and others (Supplementary Table S1) have been associated with the regulation of heart physiology in clinical studies, as well as in various animal models.

### 3.1. Identification of Densely Interconnected Genes

**Figure 3:**
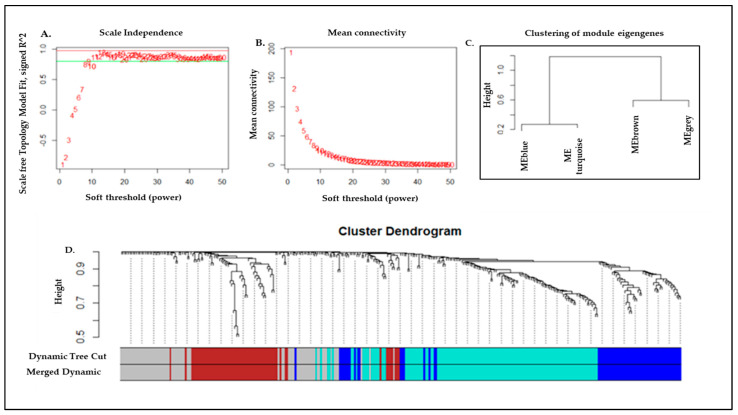
WGCNA network and module detection. Selection of the soft-thresholding powers. (**A**) the scale-free fit index versus soft-thresholding power. (**B**) The second panel displayed the mean connectivity versus soft-thresholding power. Power 91 was chosen, for which the fit index curve flattens out upon reaching a high value (>0.7). (**C**) WGCNA-derived cluster dendrogram and module assignment. Genes were clustered based on a dissimilarity measure (1-TOM). The branches correspond to modules of highly interconnected groups of genes. Colors in the horizontal bar represent the modules. Four modules with 335 annotated transcripts were detected with WGCNA. (**D**) Meta-module identification and module-module relationship. The module network dendrogram was constructed by clustering module eigengene distances. The horizontal line (blue and red line) represents the threshold (0.2) used for defining the meta-modules. Thus, 4 distinct gene modules were identified.

An average degree of connectivity and independence of co-expressed modules is highly impacted by the power value. Hence, to find the lowest power, a set of soft-threshold power values from 1 to 100 were used for Scale Free Topology Model Fit and Mean connectivity algorithms (Figure 3A,B). The network topology analysis showed the lowest power of 91, for which the scale-free topology index reaches between 0.71 (red line) and 0.60 (green line). Therefore, the threshold power value of 91 was picked up near to the curve of the plot (Figure 3). With the optimal softPower parameter (β = 91), the gene pair correlation coefficient was converted into the adjacent coefficient to calculate the dissimilarity co-expression matrix. In brief, Pearson’s correlation coefficients were calculated for genes in a pairwise manner, yielding a similarity matrix (Sij). The matrix was transformed into an adjacency matrix (aij) using a power function by formula aij  =  Power (Sij, β)  ≡  |Sij| β. Subsequently, average linkage hierarchical clustering was performed to identify modules of densely interconnected genes. Other assumptions were included: (i) the average connectivity of the distance between two classes; and (ii) ≥30 genes in each module, where a tree branch constituted one module and each leaf of the branch represented a gene. Based on these assumptions, we finally obtained 04 gene modules, namely blue, turquoise, brown, and grey (Figure 3C,D) having 67, 130, 64, and 70 genes, respectively (Supplementary Table S2). Very few genes in the blue model, such as ADORA3, AQP1, BMP5, F11R, were related to cardioprotective functions, while other genes did not show any significant association with AMI. Similarly, few genes, including DRAM2, FDFT1, AS3MT, VDAC3 in brown and grey modules, were found to be associated with AMI. For further analysis, we have selected the turquoise color gene model that was found to be carrying the highest 130 genes among all modules showing the maximum number of common genes.

### 3.2. Key Pathway Identification Using Functional Analysis for Turquoise Module

The key pathways associated with enriched genes (n = 130) in the turquoise module were identified using the STRING database. To construct the protein–protein interaction (PPI) network, STRING was used to link proteins each other (not provided as input). A PPI network of 140 nodes (genes) interacting with 116 edges showed significantly (enrichment *p*-value = 0.003) higher interactions than expected (n = 89). Using the Reactome pathway database, we extracted five significant (FDR < 0.05) pathways that were associated with the AMI disease pathophysiology. The Notch signaling pathway (hsa04330) was found to be highly associated (FDR = 2.02 × 10^−5^) with the DEGs. However, the highest numbers of DEGs were identified in the metabolic pathways (hsa01100) (Supplementary Table S3).

**Figure 4:**
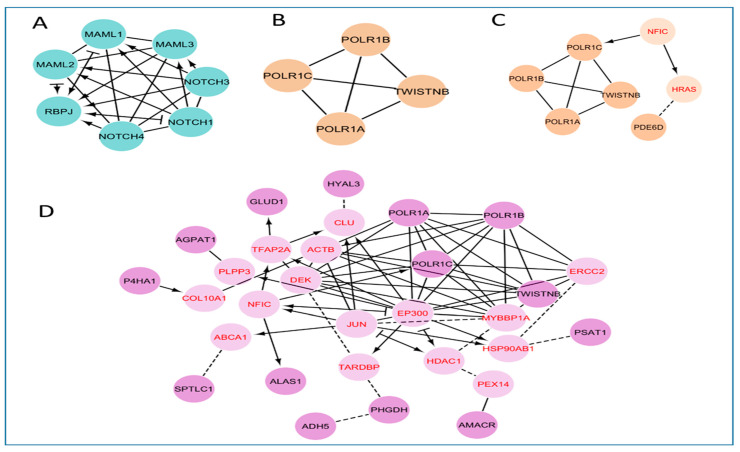
Protein–protein network analysis. (**A**) Notch signaling pathway, (**B**) Pyrimidine pathway, (**C**) Purine pathway, and (**D**) Metabolic pathways. Network edges represent Reactome functional interactions: (–) represents protein complex; (→) represents activating; (–|) represents inhibiting; (—), predicted. Clusters were labelled with the transporter classes and/or activities represented by the majority of proteins within that cluster.

Further, seven genes were found to be associated with the Notch signaling pathway (hsa04330). Of these genes, the Notch-1, -3, and -4 genes synergistically activate the other four genes, MAML-1,-2,-3, and RBPJ (Figure 4A). Five and six DEGs were found to be involved with purine (hsa00230) and pyrimidine (hsa00240) metabolism pathways, respectively (Supplementary Table S3). All DEGs other than PDE6D were the same in both the purine and pyrimidine pathways (Figure 4B,C). In the purine metabolism, out of 5 genes, 4 genes were strongly linked with each other. On the other hand, 5 genes were found to be highly connected in the purine metabolism, using 2 linker genes (NFIC and HRAS) that assist in connecting the 5th gene (PDE6D) to the network not present in the pyrimidine metabolism pathway network. In total, 14 DEGs were strongly connected to each other in the metabolic pathway (hsa01100) with the help of linker proteins. Apart from this, 16 linker genes were also involved in constructing the protein–protein network (Figure 4D).

### 3.3. Network of Genes and miRNAs of Turquoise Module

**Figure 5:**
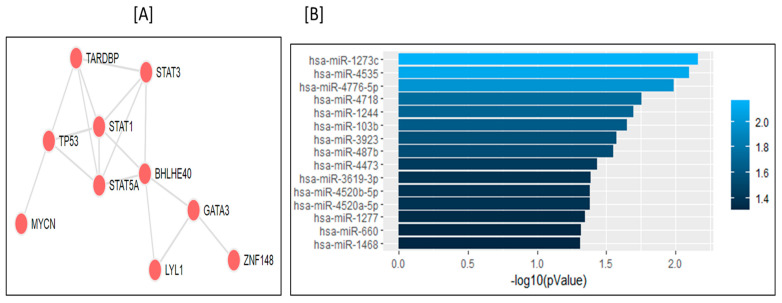
Potential factors regulating genes in the turquoise module. (**A**) Transcription factors, (**B**) Enrichment of associated microRNA.

Because the expressions of genes are regulated by transcription factors (TFs) and miRNAs, we subsequently analyzed the gene sets of the turquoise module. We found 15 significant miRNAs (*p* < 0.05; Figure 5) and STAT1 (signal transducer and activator of transcription 1) as the most important TF (Figure 5, Supplementary Table S4). These miRNAs and TFs have specific target genes, identified in the turquoise module. The identified miRNAs included hsa-miR-103b, hsa-miR-1273c, hsa-miR-4718, hsa-miR-660, hsa-miR-1468, and hsa-miR-487b, which regulate the expression of their target genes, such as BMP6, GPX3, and VPS8. (Supplementary Table S5).

## 4. Discussion

In the acute clinical setting, the detection of AMI depends on identifying necrotic cardiomyocytes, using cardiac troponin (cTn) isoforms I and T or creatine kinase MB-fraction assays along with ECG. However, annually, many patients with chest pain do not reveal these signs during admission in hospital, and only a few of them develop AMI or sudden cardiac death [25]. Hence, these conventional methods, including electrocardiogram (ECG), coronary angiography, and enzymatic indicators such as creatine kinase (CK) and creatine kinase-MB (CK-MB) isoenzyme, are associated with patient outcome in AMI [26]. However, more comprehensive assessment of the pathology underlying AMI makers is lacking, therefore this study aimed to find more specific non-traditional, as well as potential genetic biomarkers, that could identify individuals at high risk of AMI disease development and can be treated before the onset of AMI disease. In addition, these biomarker genes can be a potential therapeutic target for designing new drugs for AMI management. Furthermore, this strategy could be cost-effective, however, further experimental study is required to confirm the cost in comparison to other diagnostic methods for AMI.

Various microarray studies have been reported in AMI using bioinformatics approaches. In the microarray analysis of whole blood samples, Devaux et al. found three biomarkers that might be important in early diagnosis of AMI [27], these being vascular endothelial growth factor B (VEGFB), thrombospondin-1 (THBS1), and placental growth factor (PGF). Furthermore, while investigating three microarray datasets using WGCNA, GO and KEGG pathway enrichment analysis, a new biomarker associated with inflammations and immune response has been identified that may be involved in AMI development [28]. Later, through an integrated bioinformatics approach, Guo et al. reported 10 genes as biomarkers, including interleukin-8 (CXCL8), TNF, N-formyl peptide receptor 2 (FPR2), growth-regulated α protein (CXCL1), transcription factor AP-1 (JUN), interleukin-1 β (IL1B), platelet basic protein (PPBP), matrix metalloproteinase-9 (MMP9), toll-like receptor 2 (TLR2), and high affinity immunoglobulin epsilon receptor subunit γ (FCER1G), all of which are key genes in the pathogenesis of AMI [29]. Recently, several genes, including AQP9, IL1B, and IL1RN, have also been reported to have a potential role in AMI pathogenesis; conversely, FSTL3-miR3303p, IL1B/IL1RN, and ACSL4-miR5905p-IL1B as RNA regulatory pathways might impact AMI progression [30].

We identified 335 common annotated DEGs in the 155 samples included in all three datasets, which comprise 22 upregulated and 05 downregulated genes (*p <* 0.05) (Supplementary Table S1). Many of these genes have already been reported in AMI pathogenesis, which indicates the reliability of the integrated bioinformatics analysis results. Among upregulated genes, Bone Morphogenetic Protein 6 (BMP6), a VPS8 subunit of the CORVET complex (VPS8), Glutathione Peroxidase 3 (GPX3), B-Raf Proto-Oncogene, a serine/threonine kinase (BRAF), and an adenosine A3 receptor (ADORA3), may have an association with AMI pathogenesis.

Previous studies have suggested that adenosine (Ado), an endogenous nucleoside, is critical in protecting AMI [31]. The nucleoside Ado acts via four known receptor subtypes: A1, A2A, A2B, and A3. Of that receptor, ADOR-A3 preserves ATP (short-term) and normalizes intracellular Ca^2+^, thereby protecting cardiomyocytes from contractile dysfunction and energy depletion [16]. In the present study, ADOR-A3 was found elevated in the AMI patients group compared with the controls (Table S1), which demonstrated that the function of heart muscles is to protect themselves from ischemic damage. Normal heart functioning requires a regulated iron supply, which is tightly coupled to oxidative phosphorylation and redox signaling [32]. Notably, the iron overload has been directly correlated with cardiomyocytes apoptosis and, therefore, is a potential risk for AMI [33] with its poor prognosis [34]. Interestingly, it has been demonstrated that systemic iron homeostasis may be maintained through levels of BMP6 via Smad1/5/8 phosphorylation in the liver [35]. Since liver parameters might be pertinent factors to prognosticate the severity of stenosis, the level of BMP6 seems to contribute to AMI pathophysiology [36]. This is also supported by high levels of BMP6 in patients with advanced heart failure [37]. However, the involved molecular mechanism underlying BMP6 levels in AMI pathophysiology warrants further experimental study.

Besides metal ion balances, the protein homeostasis and signal transduction are maintained by the intracellular endolysosomal system. The VPS8 gene (along with VPS3) has been reported to regulate integrin in recycling endosomes [19], which, in a dysfunctional state, may cause several cardiac ailments and atherosclerosis [21]. In the patents filed for investigating the risk of AMI, the VSP8 gene was included in the testing panel, which hints at its role in disease pathogenesis (patent application number WO2015183601A1). However, the role of VPS8 needs further investigation in AMI development and progression.

Glutathione Peroxidase 3 (GPx3) is a selenoprotein antioxidant enzyme synthesized in the kidneys and transported to the systemic circulation [22]. The upregulated levels of GPX-3 in the cardiac tissue of diabetic mice have been demonstrated to protect cardiomyocytes against hyperglycemia-induced oxidative stress [38]. However, a reduced level of GPX3 has been associated with aging and a further elevated risk of CVDs in the elderly population [39]. The increasing incidence of CVDs has already been documented in diabetic patients, in which lower GPx3 activity might act as an independent predictor for carotid atherosclerosis [40]. The elevated levels of GPX3 in our study (Supplementary Table S1) might demonstrate its protective role in cardiac injury. Thus, this study unveils several common genes from three datasets which may act as cardioprotective biomarkers.

The development of AMI is a systemic biological process that traverses different functional networks. Weighted gene co-expression network analysis (WGCNA) is a relatively new tool to integrate and analyze several data sets and data types, such as gene expression in various cancers, and other metabolic disorders. Therefore, we have utilized WGCNA to identify the key modules and hub genes in three pooled gene expression datasets of AMI using the R package, and have identified four modules by reducing the complexity of the expression profiles. The highest numbers of genes were found in the turquoise module (Table S2). KEGG pathway enrichment and network analysis of these genes revealed their over-representation in the AMI-associated pathways. Specifically, pathway analysis of this module revealed that Notch signaling pathways, metabolic pathways, and purine and pyrimidine metabolism were the core gene sets (Supplementary Table S3). Notch signaling is a crucial mechanism underlying the normal heart morphogenetic development from embryo to adult stage [41]. In the adult heart, Notch signaling between mature cardiomyocytes is absent under normal physiological conditions, but can be reinstated as a protective response to its injury. The injury of cardiomyocytes induces the re-expression of fetal genes, leading to increased Notch1 signaling [42]. The reactivation of Notch signaling is a cardioprotective step that helps to control the extent of ischemic damage, and promotes neo-angiogenesis and revascularization of the affected cardiac tissue, thereby improving overall cardiac function [43]. In the present study, the main pathway of the turquoise module highlights Notch signaling (Supplementary Table S3, Figure 5A), in which the Notch1 gene exerts protective impact on ischemic myocardium; this is achieved via inhibiting infarct size, cardiomyocyte apoptosis, and contractile impairment in cardiac muscles via activation of PI3K/AKT pro-survival signaling [44]. Moreover, Notch signaling induces the cell cycle re-entry of immature cardiomyocytes [45]. Thus, Notch signaling in WGCNA results demonstrates another protective strategy towards normalization. Other studies have also shown different pathways and molecules in pro-survival, as well as cardio-protective roles during ischemia [46]. Furthermore, several impaired metabolic pathways have been elucidated in AMI. Altered citric acid cycle, the metabolism of glycophospholipids, **α**-linoleic acid, and sphingolipids are among the main dysregulated metabolic pathways in AMI [47].

We found various enriched miRNAs associated with our gene set of the turquoise module; these participate in AMI pathophysiology (Figure 5B) by binding to their target genes and regulating their expression. Several miRNAs have been shown to modulate various crucial pathways in cardiomyocytes and, therefore, they are potential targets for therapeutic development and predictive biomarkers. For instance, high expression levels of miR-660 elevate the generation of activated platelets, which indicates their crucial role in thrombotic events mimicking the recurrent AMI conditions [48]. The miR-660 has also been suggested to be involved in pathophysiological mechanisms that trigger recurrent MI, heart failure, and was included in panel of predictive biomarkers of AMI [49,50]. The miR-487b [51,52] and miR-1273c [53,54] have also been documented to possess prognostic value in AMI.

Moreover, there has been a widespread consensus that co-expressing genes may be co-regulated by common transcription factors (TFs); hence, we performed a gene set enrichment analysis by using the Enrichr tool [53] for the turquoise gene module. We found that STAT1 (signal transducer and activator of transcription 1) was the most significantly enriched TF (Supplementary Table S4). STAT1 participates in cardiomyocyte apoptosis by activating caspase-1 in response to IFN-γ during ischemia and reperfusion episodes [54]. Consequently, the deficiency of STAT1 could impart rescuing effects on AMI in terms of smaller infarcts, and increased levels of autophagy [55,56]. Recently, STAT1 has also been reported to have higher connectivity degrees (>20) at PPI network analysis in AMI [56]. In line with our studies, this study also identified STAT1 as a central TF that targets several crucial genes, demonstrating its potential in AMI pathology (Figure 5A).

## 5. Conclusions

In the combined microarray analysis of three datasets, a large number of biological samples from all the microarray chips capture the same information as the standard one-sample–one-chip approach. This process saves time, cost, manpower, and improves the signals. The pooled microarray analysis in the current study identified several crucial DEGs sets and miRNAs, which may be potential biomarkers in the detection and prevention of AMI. However, these results needs to be validated by experimental studies, as well the predictive values of these biomarkers in terms of their specificity, sensitivity and cost effectiveness. Importantly, we found that the Notch signaling pathway, associated with cardioprotection, could be helpful in rescuing cardiomyocytes from injury in AMI. STAT1 and miRNA (miR-660) were found to modulate the AMI pathophysiology, and thus highlight potential new targets for therapeutic development.

## Figures and Tables

**Table 1:** Characteristics of datasets included in this study.

GEO Dataset ID	GEO Platform Accession Number	Subjects		Types of Sample	Microarray Platform
		Patients	Controls		
GSE66360	GPL570	49	50	Whole blood	Affymetrix Human Genome U133 Plus 2.0 Array
GSE29532	GPL5175	8	6	Whole blood cells	Affymetrix Human Exon 1.0 ST Array
GSE62646	GPL6244	28	14	Peripheral Blood Mononuclear Cells	Affymetrix Human Gene 1.0 ST Array

**Table 2:** Genes identified from the microarray dataset may have an association with AMI.

S.No.	Name of Gene	Expression Level	References
1.	ADORA3 (Adenosine A3 receptor ADOR)	Upregulated	[15,16]
2.	AQP1 (aquaporin 1)	Upregulated	[17]
3.	BMP6 (Bone Morphogenetic Protein 5)	Upregulated	[18]
4.	VPS8 (Vacuolar Protein Sorting-Associated Protein 8 Homolog)	Upregulated	[19,20,21]
5.	(GPx3) Glutathione Peroxidase 3	Upregulated	[22,23]
6.	BRAF (B-Raf Proto-Oncogene, a serine/threonine kinase)	Upregulated	[24]

## Data Availability

Not applicable.

## Supplementary Materials

The following supporting information can be downloaded at: https://www.mdpi.com/article/10.3390/genes13122321/s1, Table S1: List of Differentially Expressed Genes (DEGs); Table S2: List of identified genes in different modules; Table S3: Enriched pathways in turquoise module; Table S4: List of identified TFs targeting genes of turquoise module; Table S5: List of significant miRNAs found targetting genes of turquoise module by Enrichr.
